# Supplementary material for: Temporal and spatial interactions in sympatric ungulates: Insights from Japanese serow and sika deer
Source: PLoS One. 2025 Aug 18;20(8):e0330171. doi: 10.1371/journal.pone.0330171 (PMC12360589; doi:10.1371/journal.pone.0330171)
Supplement: Attachment — Submitted filename: Response to Reviewers.docx [file pone.0330171.s002.docx]

Additional Editor Comments:

The reviewers have provided a comprehensive roadmap for improving the manuscript. The study is interesting but has a caveat of small sample size which could affect inferences. The implications of pooling data across years in a single season occupancy model with regard to the modelling assumptions must be elaborated. Both these shortcomings must be highlighted in the Discussion, and the Abstract should include a disclaimer on small sample size. Should you decide to revise the manuscript and submit the revision to the journal, please address all comments from the reviewers point by point, either by incorporating in a revised manuscript or by rebutting the request.

***Response***: Thank you for your comments and for providing a clear roadmap for improving our manuscript. We appreciate the reviewers’ valuable feedback and have carefully addressed each of their concerns. Regarding the small sample size, we have explicitly acknowledged its potential limitations in the Discussion and included a disclaimer in the Abstract, as recommended. We also have elaborated on the implications of pooling data across years in a single-season occupancy model, ensuring that the modeling assumptions are properly discussed. For a detailed account of how each reviewer’s comment was addressed, please refer to our point-by-point responses to the reviewers. Additionally, we have completely revised Figure 1 to comply with the required guidelines. The new figure no longer contains any copyrighted materials and we have updated the figure legend to reflect this revision as follows:

“We created this map ourselves using data provided by the Geospatial Information Authority of Japan (https://www.gsi.go.jp/) and Biodiversity Center of Japan (http://gis.biodic.go.jp/webgis/), under the CC BY 4.0 or a compatible license.”　(Line 147-150)

We appreciate the opportunity to revise our manuscript!

**Reviewer #1:**

**Major comment**: The authors assess whether the increase in sika deer population may influence the behavior and presence of the Japanese serow. I find this topic very interesting, and it seems timely given the rapid expansion of one species. The manuscript is overall well-structured and provides a basic understanding of the conducted research and the conclusions drawn from it. While I believe the statistical methods are applied appropriately, the underlying assumptions are somewhat problematic. This is primarily due to the lack of information provided in the methods section.

In the discussion the authors mention that the deer population is probably too low to have an effect on serow. This seems to be existing knowledge and not derived from this investigation. This is a key factor and an explanation for many of the discussed aspects earlier on. I suggest to provide this information earlier and explain why the research is still valid and important despite this.

***Response:*** We sincerely appreciate the reviewer's time and effort in evaluating our manuscript, as well as their positive feedback. We also acknowledge the concerns regarding the underlying assumptions and the need for additional methodological details. In response to these comments, we have revised the Methods section to provide further clarification on data collection and analytical assumptions.

Additionally, we recognize that the current sika deer population density in our study area may be lower than in other regions where stronger interspecific competition has been observed. As this is a key factor influencing our findings, we have introduced this information earlier in the Introduction, explaining why studying interactions at this stage remains valid and important. Understanding the behavioral responses of Japanese serow under relatively low deer densities provides crucial insights into potential long-term impacts as deer populations continue to expand. In the Discussion, we further emphasize that our findings should be interpreted within the context of deer population density and highlight the importance of ongoing monitoring to assess future competitive interactions as deer densities increase.

Please see more details in our responses to your specific comments below.

Furthermore, the manuscript contains many grammatical errors and shows signs of insufficient attention to detail. This makes reading the manuscript somewhat cumbersome and I suggest that the authors consult a copyeditor. At this point, I do not believe the manuscript is ready for publication but I would not reject it outright and rather provide the opportunity for revisions.

***Response:*** According to your comments, we have consulted a professional English editing service for thorough proofreading and refinement as stated in the acknowledgments.

Abstract

**Comment#1 (Line 28)**: Not needed: ...although they coexisted…

***Response:*** According to your comment, we have removed the phrase "although they coexisted" to improve conciseness as follows:

From: “In Japan, since the 1970s, the endemic Japanese serow (*Capricornis crispus*) and the sika deer (Cervus nippon) were primarily allopatric, although they coexisted in localized regions.”

To: “In Japan, since the 1970s, the endemic Japanese serow (*Capricornis crispus*) and the sika deer (*Cervus nippon*) have been primarily allopatric.”. (Line 26-28)

**Comment#2 (Line 31)**: What are the potential impacts?

*Response*: We have clarified the potential impacts by specifying concerns about interspecific competition for food and habitat resources, which may influence the distribution and behavior of both species as follows:

From: “This increasing overlap raises concerns about the potential impacts between these two sympatric ungulates.”

To: “The significant and increasing overlap raises concerns about the potential impacts between these two (now sympatric) ungulates, including changes in distribution, shifts in activity patterns, or displacement due to interspecific competition.” (Line 29-32)

Introduction

**Comment#4**: The introduction is largely well-written and provides a good introduction to the two species and their potential interaction. However, I feel it would benefit from a clearer lead-up to the specific study area and a more explicit explanation of why investigating these two species in this context is important. Are there any broader implications for the ecosystem or for the species themselves? Are there any direct management implications that can be derived from this manuscript? Furthermore, I suggest starting with an explanation or definition of the term sympatric. While it is widely used and most readers are likely familiar with it, the term is central to this paper and should be clarified to avoid any potential confusion. For instance, in lines 57 to 59, are zebras and wildebeests truly sympatric, or are they simply more sympatric compared to other animals in this ecosystem?

I started by pointed out grammatical errors, typos, and formatting issues but I will refrain from doing so throughout the manuscript.

***Response:*** We appreciate the reviewer’s constructive feedback on improving the clarity and structure of the Introduction. According to your comments, we have revised the Introduction to provide a clearer lead-up to the study area, explicitly highlighting why investigating the interactions between these two species in this context is important. We now emphasize the conservation importance of the Japanese serow, including its designation as a "Threatened Local Population" in certain regions, and the management challenges posed by increasing sika deer populations. Additionally, we have clarified the term “sympatric” at the beginning of the Introduction to ensure consistency in its usage throughout the manuscript. We have revised the entire introduction. Please check it.

Added: “Sympatric species are those that coexist in the same geographic area, often sharing similar ecological niches, which creates the potential for competition over limited resources such as habitat and food [1].” (Line 52-54)

More detailed comments:

**Comment#5** (Line 59): I recommend double-checking this reference as to my understanding the authors representation do not fully align with the outcomes of Fuller et al. Additionally, this example could be explained somewhat more, similar to the other two given in this context.

***Response:*** According to your comments, we have re-reviewed the reference by Fuller et al. (1989) and found that our previous description did not fully consistent with their findings. We have revised the text to ensure that our representation accurately reflects the study’s outcomes as follows:

From: “In Kenya, three sympatric jackal species exhibit distinct diurnal activity patterns”

To: “In Kenya, three sympatric jackal species partition their activity times, with golden jackal (*Canis aureus*) being active during the day, while black-backed jackal (Lupulella mesomelas) and side-striped jackal (*L. adusta*) are mostly nocturnal”	(Line 64-66)

**Comment#6** (Line 69): Selective diet not diets?

***Response:*** According to your comment, we have revised "selective diets" to "selective diet" to ensure grammatical accuracy as follows:

From: “＿＿＿, and has a relatively narrow, selective diets”

To: “______, and has a relatively narrow, selective diet”	(Line 79)

**Comment#7** (Line 69): Inhibits alpine meadows not inhibits in alpine meadows?

***Response:*** According to your comment, we have revised "inhabits in alpine meadows " to "inhabits alpine meadows" to ensure grammatical accuracy as follows:

From: “. It also inhibits in alpine meadows, ______”

To: “It also inhibits alpine meadows”	(Line 79-80)

**Comment#8** (Line 73): I believe the species can only be either clearly allopatric or partly sympatric, as these are distinct concepts. Rephrasing this would help improve clarity.

***Response***: According to your comment, we have clarified that the two species were generally allopatric, but there were a few localized exceptions in northern Japan, as follows:

From: “Until the 1970s, these species were clearly allopatric, although deer were also distributed in northern Japan and they partly inhabited sympatrically [14,15]”

To: “Until the 1970s, these species were allopatric, except in a few isolated areas in northern Honshu where their distributions overlapped [15,16]”	(Line 83-84)

**Comment#9** (Line 72-75): What occurred between 1970 and 1990? Why did the authors choose to report specifically on these two dates?

***Response:*** We selected the period between 1970 and 1990 because it marks a transitional phase in the population dynamics and distribution of sika deer in Japan. Until the 1970s, their populations were relatively stable and confined to certain regions due to historical overhunting and habitat loss. However, since the 1990s, the population has increased significantly, with a dramatic expansion in their range [16], likely due to factors, such as reduced hunting pressure, land-use changes, and milder winters. According to your comments, we have revised the text to clarify this transition and provide context for our choice of these time periods."

From: “However, since the 1990s, the deer population has increased and their distribution (including elevation) has expanded dramatically [16]”

To: “However, since the 1990s the deer population has increased and its distribution (including elevation) has expanded dramatically [17], driven by factors such as reduced hunting pressure, changes in land use, and milder winters [18].” (Line 84-87)

**Comment#10** (Line 89-91): This sentence needs rephrasing, as it currently implies that the behaviour is solely caused by autumn, whereas the focus is on autumn being the mating season.

***Response:*** According to your comment, we have revised the sentence to clarify that the increase in local deer density and their heightened activity are due to mating-related behaviors in autumn, rather than the season alone as follows:

From: “Furthermore, the competitive relationship between these two species may vary depending on the season. Since deer in Japan mate in autumn, males congregate at mating sites and establish harems of several females each [16], thereby leading to a temporary rise in their local population density.”

To: “Furthermore, the competitive relationship between these two species may vary depending on seasonal changes in behavior. During the autumn mating season, male deer congregate at mating sites and establish harems of several females each [17], thereby leading to a temporary rise in their local population density.” (Line 109-112)

**Comment#11** (Line 80-98): This section helps me to understand the relationship between the two species and the significance of autumn, well done. However, in the discussion the authors mention that deer are clearly more dominant than serow. I believe this should be outlined in this section already.

***Response***: We agree that outlining the dominance of deer over serow earlier in the manuscript would improve clarity and strengthen the logical flow of the discussion. According to your comment, we have explicitly stated that deer are generally dominant over serow, highlighting their larger body size, aggressive rutting behavior, and ability to outcompete serow for resources as follow:

　　　Added: “Deer can be considered to have a competitive advantage over serow due to their larger body size, social behavior, and broad diet [20–22]. Their ability to exploit a wider range of food resources allows them to outcompete the more specialized serow, particularly in resource-limited habitats [21]. Furthermore, some studies report that serow avoid deer in areas with high deer density [22]. Although serow occasionally display territoriality behaviors toward deer, they rarely succeed in displacing them [23]. Overall, the combination of a larger body size, higher population density, and greater dietary flexibility is thought likely to provide sika deer with a long-term competitive edge in sympatric habitats.”

(Line 91-99)

**Comment#12** (Line 102-104): How did it prove ideal for the study species? I suggest eliminating this sentence but if it is important and indeed the ideal choice of method, the authors should explain why.

***Response:*** According to your comment, we have deleted this sentence.

Deleted: “Camera trapping is a widely-used effective and non-invasive tool for studying wildlife [21], which proved ideal for our study area and study species.” (Line 124)

Materials and methods

**Comment#13:** I believe the statistical methods are applied correctly and can be used to address the research question. However, I am uncertain whether the underlying assumption of pooling data over multiple years into a single-season model is valid, especially if the locations are not constant. To provide final feedback, I would need more detailed information about the study setup. Please refer to my comments below for further clarification.

Response: Thank you for your valuable feedback and for recognizing the appropriateness of our statistical methods in addressing the research question. We appreciate your concern regarding the assumption of pooling data over multiple years into a single-season model, particularly in relation to location consistency.

To address your concern, we provide additional details on our study setup and data collection methodology. We have carefully reviewed your subsequent comments and provided detailed responses to each of them. Please find our point-by-point responses below for your review.

**Comment#14:** Figure 1 should be improved by adding more details, such as clearly marking the borders of the national forest, using more easily distinguishable symbols for the camera traps per year, and especially providing a higher-resolution image. Additionally, the caption should be revised to offer a more thorough and descriptive explanation of the figure. It appears that sampling occurred very close to a human settlement, which contradicts the earlier statement that there was little human disturbance. Furthermore, the figure suggests a spatial separation of locations, while the text states that multiple sites were sampled repeatedly. Please provide clarification.

**Comment#15** (Line 116): The map does not indicate the boundaries of the national forest, and it would be important to communicate what proportion of the study area is within the national forest. A short explanation what a national forest is would be good too. Furthermore, how do the authors assess the level of human disturbance? Finally and very importantly, what fraction of the survey effort was conducted in each of these areas?

***Response:*** To improve Figure 1, we have enhanced its resolution, updated the symbols for camera trap locations to improve clarity, and revised the figure caption for better detail. We have also removed the reference to the "national forest," as this classification is specific to Japan’s forest management system and is not particularly relevant to our study; therefore, we did not include its boundaries or proportion within the study area.

We consider human disturbance at our study sites to be relatively low for the target species for the following reasons. First, all survey sites were at least 600 m from the town ridge, reducing direct human influence. Ikeda et al. (2022) found that sika deer are more frequently detected in areas with lower human disturbance, using a 500 m buffer, suggesting that our study sites, located over 600 m from human settlements, experience limited human impact. Second, Japanese serows are generally less sensitive to human activity than sika deer (Ikeda et al. 2022). Third, in the case of 2017, no sika deer were captured during our survey period (excluding the hunting season), and only a few were captured outside this period (Gifu Prefecture, 2023), further minimizing human influence.

Considering this, we have revised the entire Study Area section and Figure 1.

Please refer to the main text and Figure 1 for details.

**Comment#16** (Line 121): Is this relevant given that the study was conducted only between 800 m and 1600 m? Or did I misunderstand something? If it is only for the sake of completeness I suggest adding information about the area above 1600 m too.

**Comment#17** (Line 118 and 125): First, it is mentioned that 12% of the area are cedar plantations, but it remains unclear how the other 88% look like. Then, cedar is mentioned again in line 125, is this a repetition or an oversight, or do the authors refer to something else, please explain?

***Response:*** Sorry. Our study was conducted at elevations between 600 m and 1,600 m, not between 800 m and 1,600 m. Therefore, we have focused only on the vegetation below 1,600 m and have removed references to vegetation above this elevation. Additionally, we clarified that forestry plantations, predominantly consisting of Japanese cedar (*Cryptomeria japonica*), account for approximately 12% of the entire area and are mostly distributed below 1,600 m in a patchy manner. We also removed redundant mentions of forestry plantations to improve clarity and avoid repetition.

From: “Forestry plantations, predominantly consisting of mature Japanese cedar (Cryptomeria japonica), comprise approximately 12% of this area [22]. The local area has a humid continental climate, with warm, humid summers (averaging 21.8℃ from 2015–2017), and cold, snowy winters (averaging 0.9℃ over the same period).

The forest vegetation below 800 m consists of broad-leaved trees, such as konara oak (*Quercus serrata*) and Japanese chestnut (*Castanea crenata*). Above 800 m but below 1,600 m, Mongolian oak (*Q. crispula*) and Japanese beech (*Fagus crenata*) predominate.”

To: “It is characterized by steep, rugged terrain with deep valleys and is predominantly covered with broad-leaved forests, with approximately 12% consisting of patchily distributed Japanese cedar (*Cryptomeria japonica*) plantations at elevations below 1,600 m [27]. The local area has a humid continental climate, with warm, humid summers (averaging 21.8℃ from 2015–2017), and cold, snowy winters (averaging 0.9℃ over the same period). The forest vegetation below 800 m consists of broad-leaved trees, such as konara oak (*Quercus serrata*) and Japanese chestnut (*Castanea crenata*). Above 800 m but below 1,600 m, Mongolian oak (*Q. crispula*) and Japanese beech (*Fagus crenata*) predominate.” (Line 137-154)

**Comment#18** (Line 127): How did the authors conclude that there was no degradation of vegetation and food was considered plenty if no data was collected or a reference given? I think some further explanation is needed here.

***Response:*** It has been found that the evaluation based on the Shrub-layer Decline Rank (SDR, Fujiki et al. 2010, J. For. Res.) indicates minimal impact of deer-induced understory vegetation decline in the vicinity of the study site (Gifu Prefecture 2021). Therefore, we have revised the text as follows:

From: “There was no observable degradation of vegetation in the study area during the study, and food resources for both serow and deer were considered to be abundant (although specific data on food availability was not collected) (Fig. 1).”

To: “The shrub-layer decline rank evaluation [SDR, 28] in 2016 indicated minimal deer-induced vegetation decline in the study area [29], indicating no observable degradation of vegetation in the study area during the study, and food resources for both serow and deer were considered to be abundant.”

(Line 155-158)

**Comment#19** (Line 136): Please explain here that it was only 39 cameras in total over 3 years. This is key information and should be stated clearly.

***Response:*** We have clarified in the text that a total of 39 camera traps were deployed over the three-year period to ensure this key information is explicitly stated as follows:

From: “Over a period of three years (2015–2017), we installed 39 camera traps in various habitats at various elevations to ensure a comprehensive representation of the local environment.”

To: “Over a period of three years (2015–2017), we installed 39 camera traps (14 in 2015, 11 in 2016, and 14 in 2017) in various habitats at various elevations, to ensure a comprehensive representation of the local environment.” (Line 175-177)

**Comment#20** (Line 137): The authors state that cameras were placed in three habitat types to ensure comprehensive representation. However, it seems that the habitat is dominated by a single vegetation type. Based on the provided information, it is difficult to evaluate this clearly, see my previous comments. This lack of information is one of my main concerns with the methods and results of this manuscript.

**Comment#21** (Line 139): I thought the area primarily consisted of broad-leaved forests but here the authors mention coniferous forest and riparian forest.

***Response:*** We acknowledge the reviewer’s concerns regarding habitat classification and the dominant vegetation type. While the study area is primarily composed of broad-leaved forests, coniferous forests and riparian forests are also present. To clarify this point, we have revised the text to explicitly describe the presence of each habitat type as follows.

From: “Camera traps were placed within three broad vegetation types (broad-leaved forest, coniferous forest, and riparian forest).”

To: “Camera traps were installed based on the survey routes established by Mori et al. [33] and their surroundings, as well as other key locations, proportionally distributed across vegetation types: broad-leaved forest, plantations, and riparian forest located along streams and rivers.” (Line 188-191)

Additionally, we have revised Figure 1 to indicate the specific locations within the study area where the environmental photos were taken. Please check Figure 1.

**Comment#22** (Line 140): Does this timeframe ensure demographic closure?

***Response:*** We acknowledge your concern regarding demographic closure. The study period was set after the birthing season, ensuring that population fluctuations due to new births were minimal. While deer are known to exhibit seasonal movements, the study was conducted before snowfall, minimizing the likelihood of such movements. Considering these, we have added the following sentences:

Added: “The study period was set after the birthing season (mainly May to June: [17]), minimizing population fluctuations due to new births. While deer are known to exhibit seasonal movements in regions with heavy snowfall [31,32], the study was conducted before snowfall, reducing the likelihood of such movements. Therefore, demographic closure was likely maintained during the study period.”

(Line 178-182)

**Comment#31** (Line 183): I kept wondering about home range sizes for the species, thanks, please give more details.

**Comment#23** (Line 140-143): Please provide more details on the spatial placement of these cameras within the study area.

**Comment#25** (Line 146): The distance seems to vary greatly please elaborate further on how all of those ensure independence?

***Response:*** According to your comments, we have included a more detailed description of the camera trap placement strategy. Cameras were positioned along survey routes established by Mori et al. (2017) and surrounding areas, as well as other key locations, with placement proportional to the distribution of vegetation types (e.g., more cameras were placed in dominant vegetation types). The minimum distance between camera traps was set at 500 m to reduce spatial dependence. However, in some cases, terrain constraints required shorter distances between some cameras. Considering the home range sizes of the target species, independence was largely maintained, though it is possible that the same individual was recorded by adjacent cameras.

We have added the explanation in the text as follows:

Added: “Camera traps were installed based on the survey routes established by Mori et al. [33] and their surroundings, as well as other key locations, proportionally distributed across vegetation types: broad-leaved forest, plantations, and riparian forest located along streams and rivers. The average inter-camera distance was 1,028 ± 602 m, though terrain constraints resulted in some placements as close as ~350 m. Given the home range sizes of serow (0.16–1.62 km2: [10]) and deer (0.19–2.59 km2: [32,34]), independence was largely ensured, though some individuals may have been recorded by multiple cameras. To capture a wide range of environmental conditions, cameras were also distributed across different topographic features, including valleys, ridges, and slopes. These vegetation and topographic variations were considered to assess potential differences in habitat use by the two focal species”

(line 188-199)

**Comment#24** (Line 145): Which ones were placed at the same location? Maybe a table would help here.

***Response:*** Instead of adding a table, we have improved the figure to more clearly indicate which camera traps were placed at the same locations across the years. Please check the Figure 1.

**Comment#26** (Line 166): I believe there is a comma missing after (p), or I do not follow the sentence completely.

***Response:*** Thank you! According to your comment, we have added a comma after (p) as follows:

From: “We employed occupancy models [28] to estimate occupancy probability (ψ) and detection probability (*p*) and to analyze co-occurrence patterns between deer and serow.”

To: “We employed occupancy models [28] to estimate occupancy probability (ψ) and detection probability (*p*), and to analyze co-occurrence patterns between deer and serow.” (Line 220-221)

**Comment#27** (Line 167/168): Is this information relevant?

***Response:*** No. We have deleted the sentence.

Deleted: “Occupancy models have been widely used in camera-trap studies to determine wildlife species occurrence and distribution [28]”	(Line 222)

**Comment#28** (Line 167-173): I do not believe this detail is needed, a suitable reference should explain this sufficiently, and a simple sentence would be enough for this manuscript.

**Comment#30** (Line 179 -180): How does an occupancy model determine the habitat characteristics of a species? By providing occupied areas per habitat?

***Response:*** We agree that a detailed explanation is unnecessary and that a suitable reference can sufficiently support the information. We have revised the text to provide a more concise statement while citing an appropriate reference as follows:

From: “These models utilize data collected from repeated surveys to construct detection histories, which record the presence or absence of a species at each sampling location over multiple sampling occasions, providing an unbiased estimate of the probability that an area (the camera-trap location) is occupied by a species when detection probability (p) is < 1. Furthermore, these models can incorporate covariates to analyze their effects on ψ and p.”

To: “These models use detection histories from repeated surveys to estimate occupancy probability (ψ) and detection probability (p) and can incorporate covariates to assess their effects [40]. Occupancy models do not directly determine habitat characteristics but estimate the probability of a species occupying a given site while accounting for imperfect detection.” (Line 222-226)

**Comment#29** (Line 174-176): This not clear to me. It seems that only a few camera trap locations were sampled repeatedly, so the dataset should be reduced to include only those specific locations? Is it justified to pool these locations, especially if they were not identical, as suggested by the figure? I would appreciate more information that convinces me that this approach is justified. Additionally, the mention of six sampling occasions of 10 days seems odd, considering that the cameras were operating for four months each year. Did the authors reduce the dataset based on the 'early, middle, and late periods of each summer and autumnal month'? This phrasing is unclear to me.

***Response:*** Thank you for your insightful comment. We pooled data across years and incorporated "year" as a covariate in a single-season occupancy model, which is appropriate when different locations are sampled each year within a consistent study area. This approach allows us to estimate annual variation in occupancy while maintaining sufficient sample size. Although a multi-season model could be used, we did not aim to estimate colonization and extinction, making the single-season model with year as a covariate a more suitable choice.

A Multi-season model is only necessary when the same locations are sampled across years, allowing estimation of colonization and extinction rates. In our study, camera trap locations varied between years, although we maintained consistent coverage of the study area's major habitat types (broad-leaved, coniferous, and riparian forests). Given this design, applying a multi-season model would be inappropriate because our objective was not to track site-level changes over time but rather to estimate annual differences in occupancy at a broader scale***.*** Considering this, we have added the explanation regarding this as follows:

Added: “We pooled data across years and incorporated "year" as a covariate in a single-season occupancy model, which is appropriate when different locations are sampled each year within a consistent study area. This approach allowed us to estimate annual variation in occupancy while maintaining sufficient sample size. Although a multi-season model [42] could have been used, we did not aim to estimate colonization and extinction, making the single-season model with year as a covariate a more suitable choice.”

(Line 241-246)

Additionally, for each of the three study years, each season (summer: July–August, autumn: September–October) was divided into early (days 1–10), middle (days 11–20), and late (days 21–30) periods per month, capturing potential shifts in species activity while maintaining full data coverage. Although months differ in length (30 or 31 days), we standardized all months to 30 days for consistency, removing the 31st day where applicable. To clarify this, we have revised the manuscript as follows:

From: “In our study, we used six sampling occasions of 10 continuous days each (early, middle, and late periods of each summer and autumnal month.”

To: “In our study, we conducted six sampling sessions, each lasting 10 consecutive days, covering the early (days 1–10), middle (days 11–20), and late (days 21–30) periods of each summer and autumn month (July–October). To ensure consistency across years, all months were standardized to 30 days by excluding the 31st day. Data were pooled across three years (2015–2017), resulting in 39 camera trap locations.”

(Line 227-231)

**Comment#32** (Line 184): Which assumptions?

**Comment#33** (Line 185): This clarifies my point from line 179-180, please mention this earlier.

***Response:*** According to your comments, we have added information on the home range size of the target species in the manuscript (around line 185-187) and have revised the text regarding the assumptions as follows:

From: “Since the home ranges of the two species being studied may have exceeded the area covered by our sampling sites, we relaxed these assumptions by interpreting “occupancy” as “habitat use”, as proposed by [30].”

To: “The camera spacing generally accounted for home ranges, but some were placed closer together, potentially exceeding the species' ranges. Therefore, we followed Mackenzie et al. [40] in interpreting “occupancy” as “habitat use.” (Line 238-240)

**Comment#34** (Line 186): Covariates not covariances?

***Response:*** Thank you for pointing that out. It was a typo. So, we have revised the text as follows:

From: “For covariances were used to assess…”

To: “Four covariates were used to assess…”	(Line 246)

**Comment#35** (Line 196): What justifies keeping detection probability constant?

***Response:*** Thank you for your comment. We assumed a constant detection probability for the following reasons. First, our sample size was relatively small (n = 39), and holding detection probability constant helped avoid overparameterization. Second, all camera traps were deployed using a standardized protocol, with consistent settings, heights, and angles. Third, vegetation in front of the cameras was cleared as necessary to maintain visibility, reducing potential differences in detection probability between cameras. Finally, the camera deployment period was the same each year, ensuring consistency across survey years.

Considering this, we have revised the text as follows:

From: “When building the models, we held the detection probabilities constant.”

To: “When building the models, we assumed that detection probabilities remained constant for the following reasons. First, our sample size was relatively small (n = 39), making it necessary to limit the number of parameters to avoid overparameterization. Second, all camera traps were deployed using a standardized protocol, maintaining consistent settings, heights, and angles. Third, vegetation in front of the cameras was cleared as necessary to maintain visibility, minimizing potential differences in detection probability between cameras. Finally, the camera deployment period remained the same each year, ensuring consistency across survey years.” (Line 255-264)

**Comment#36** (Line 217): Subordinate because not territorial?

**Comment#37** (Line 223-224): This information should be provided earlier on.

**Comment#45** (Line 291): So deer presence should have influenced sika as deer is more dominant? If this is clear than it should be presented earlier.

***Response:*** Thank you for your comments. We have addressed the competitive advantage of sika deer in the Introduction, as mentioned in my previous response. We believe this adequately addresses the concern raised in this comment.

Results

**Comment#38:** I think the results are well presented and appear logical. However, my concern remains with the modelling assumptions.

Response: Thank you! Regarding the modeling assumptions, we have addressed these concerns in our previous responses to your comments**#29**.

**Comment#39** (Line 250): This sentence could be improved for increased readability.

　 　Response: Thank you for your suggestion. To improve readability, we have restructured the sentence for clarity and conciseness.

From: “Serow exhibited cathemeral activity with multiple peaks in summer, tending towards dawn, but their activity pattern shifted to nocturnal behavior in autumn (Fig. 2). In contrast, deer were crepuscular, with activity peaks during the crepuscular periods during summer and autumn, but tending towards dusk in summer and towards dawn in autumn (Fig. 2). Furthermore, deer also demonstrated a relatively high level of daytime activity.”

To: “Serow exhibited cathemeral activity in summer, with multiple peaks centered around dawn. In autumn, their activity shifted to a predominantly nocturnal pattern (Fig 3). In contrast, deer were crepuscular, showing activity peaks during twilight hours in both summer and autumn. However, their activity tended towards dusk in summer and towards dawn in autumn (Fig 3). Additionally, deer displayed a relatively high level of daytime activity.” (Line 317-322)

**Comment#40** (Table 1): Could this data be presented in a figure? I think this is very important information and a figure is easier to interpret.

Response: Thank you for your insightful suggestion. In response, we have transformed Table 1 into Figure to enhance clarity and facilitate interpretation. Please check Figure 2.

Discussion

**Comment#41:** I suggest structuring the discussion in the same order as the methods and results sections, following the same sequence of topics.

***Response:*** Thank you for your valuable suggestion. According to your recommendation, we have restructured the discussion section to follow the same sequence as the methods and results sections, specifically in the order of RAI, Temporal activity patterns, and spatial distribution and habitat preferences. Additionally, to enhance clarity, we have added subheadings to each section. This helps to organize the discussion more effectively and allows readers to follow each topic more easily. However, since both temporal and spatial perspectives are necessary to fully understand the interactions between sika deer and Japanese serow, we also have introduced a new subheading, "Implications for competition and coexistence". This section provides a more comprehensive discussion on the relationship between the two species, helping to clearly highlight the key implications of our findings.

**Comment#42** (Line 288): I believe it had three vegetation types even though that was never clearly explained and I am not sure about the habitat types

***Response:*** According to your **comments#23** and **#25**, we have provided more details on the camera locations, including vegetation. Please refer to our replies to your **comments#23** and **#25** for details.

**Comment#43** (Line 289): I disagree with the statement that "any potential bias is minimized," as I believe this is an overstatement. The authors seem to acknowledge this by using careful language such as "cautiously interpreted" but I would suggest rephrasing the first part of the statement.

**Comment#44** (Line 290): Without providing more detail on site selection I do not see how this can be the case.

***Response:*** Thank you. According to your comments, we have rephrased the statement as follows:

From: “Although our study was conducted on a relatively small scale, the study area incorporated a wide range of vegetation and habitat types representative of larger regions. Therefore, we believe that any potential bias due to scale was minimized, and our findings may be cautiously considered valid even when applied to broader areas.”

To: “Our study was conducted within a limited spatial range, and the relatively small sample size (n = 39) poses a limitation that must be acknowledged. While efforts were made to mitigate potential bias, the results should be interpreted with caution when applied to broader contexts.” (Line 376-379)

**Comment#46** (Line 293-95): Can the authors think of any other explanation for a reduction in the overlap of activity times in autumn that might have to do with food availability or hunting?

**Comment#47** (Line 299/302): I believe hunting can influence wildlife movement even outside the hunting season. Given the proximity of a village and the apparent presence of hunting, I am not sure it is accurate to say that there is minimal impact. Additionally, the survey stopped just 15 days before the start of the hunting season, and this seems to be an annual occurrence. More information would help to convince the reader that the impact of hunting and human presence, both during and outside the season, has no impact.

***Response:*** As stated in our previous response (your **comment#14,15**), hunting is expected to have minimal impact in our study area. While no studies in Japan have directly linked seasonal vegetation changes (food availability) to shifts in activity patterns of these species, previous research on sika deer and Japanese serow in other regions of Gifu Prefecture has demonstrated that their diel activity patterns do not change seasonally (Ikeda et al. 2022). This suggests that food availability is unlikely to be the primary driver of the observed seasonal changes in activity overlap.

Considering these, we have added the following sentences in the text.

Added: “While seasonal shifts in food availability could influence activity patterns, previous research in Gifu Prefecture indicates that sika deer and Japanese serow do not exhibit seasonal changes in diel activity [47], suggesting that food availability is unlikely to be the primary factor. Additionally, human disturbance is expected to be minimal in our study area. Therefore,” (Line 396-400)

**Comment#48** (Line 312): Now the authors speak of other areas with less human disturbance, while it was stated numerous times already that there is little disturbance in this study system? Please address this inconsistency.

***Response:*** Thank you for your suggestion. According to your comments, we have revised the manuscript to ensure consistency in describing human disturbance within our study area as follows:

From: “We found deer to be diurnal, both in summer and autumn, which is consistent with previous studies in regions with less human disturbance [39,43,44]. Similarly, red deer (*C. elaphus*), mule deer (*Odocoileus hemionus*), and white-tailed deer (*O. virginianus*) display crepuscular activity patterns [45,46,47]. This activity pattern is a common deer trait, motivated by predator avoidance and heat stress mitigation [48,49,50]. This might be because deer in Japan have lacked predators since the extermination of Grey Wolf (*Canis lupus*) over 100 years ago [51]. Thus, their current combination of crepuscular and diurnal activity may allow deer in Japan to optimize their behavior for both survival and resource acquisition.”

To: “We found deer to be diurnal, both in summer and autumn, which is consistent with previous studies in regions with less human disturbance [52,56,57]. Similarly, red deer (*C. elaphus*), mule deer (*Odocoileus hemionus*), and white-tailed deer (*O. virginianus*) display crepuscular activity patterns [58–60]. Predation risk is a key factor influencing diel activity in many deer species. However, in Japan, large predators have been absent since the extermination of the gray wolf (*Canis lupus*) [60]; thus, human disturbance may have a greater influence on deer behavior [61]. However, since our study area experiences minimal human disturbance, the observed diurnal or crepuscular activity in sika deer is likely a natural behavioral pattern rather than a response to human influence.” (Line 421-429)

**Comment#49** (Line 311): Please stick to deer and not sika deer to avoid confusion.

***Response:*** Thank you. According to your comment, we have replaced "sika deer" with "deer" throughout this section to maintain consistency and avoid confusion.

From: “We found sika deer to be diurnal, both in summer and autumn, …”

To: “We found deer to be diurnal, both in summer and autumn, …”	(Line 421)

**Comment#50** (Line 326-329): I do not think that listing these options is very convincing, I would prefer to be provided with some fewer options but have those explained more, similar as to what was done in the next sentence.

***Response:*** According to your comments, we have revised this section by reducing the number of listed factors and providing a more detailed explanation for each as follows:

From: “The discrepancy between our findings and those of previous studies might arise from factors, such as varying degrees of human disturbance, different vegetation types, resource availability, or reduced competition in our study area. Our study area is distinctive in that it encompasses a significant portion of national forest, with few plantations, and experiences minimal human disturbance, which may affect the habitat preferences of both species. Further research is essential to clarify these inconsistencies.”

To: “The discrepancy between our findings and those of previous studies might arise from factors, such as varying degrees of human disturbance, different vegetation types, or resource availability. In areas with high human disturbance, habitat conditions may change, potentially influencing the behavior and distribution of deer and serow. In contrast, in regions with minimal human impact, their natural habitat preferences may be more clearly expressed. Another factor could be differences in vegetation and food availability. Areas dominated by broad-leaved forest may provide more suitable habitat for serow, whereas areas rich in herbaceous plants may offer more favorable conditions for deer. Our study area is distinctive in that it encompasses a significant portion of broad-leaved forest, with few plantations, and experiences minimal human disturbance, which may affect the habitat preferences of both species. Further research is essential to clarify these inconsistencies.” (Line 438-450)

**Comment#51** (Line 344-346): If this is clear I am wondering if the outcome of this investigation could have not been predicted by this circumstance. In fact if this the case does it not make most other assumptions redundant?

***Response:*** Thanks. As mentioned in our previous response to the Major comment, we have added an explanation in the Introduction to clarify why studying species interactions under these conditions remains valid and important. Please review the revised Introduction.

**Comment#52** (Line 347-352): This is very well explained and makes sense to me! As such the inference drawn in line 355 is key in my eyes. Would that not be an explanation for many of the things that have been discussed so far too?

***Response:*** Thank you!

**Comment#53** (Line 369-373): I thought the deer density is not high enough to have an effect?

***Response:*** Thank you for your comment. Our study area experiences minimal human disturbance, and the influence of food resource availability is also considered to be limited. Therefore, the observed shift in serow activity patterns is likely due to the temporary increase in deer density during the rutting season.

As mentioned in our response to your **comment #47**, we have emphasized this point at the beginning of the discussion section to clarify its significance.

Reviewer #2: Compliments on an appropriate use of a small data set, to address your hypothesis. Please see my detailed comments below for improving your manuscript -

***Response:*** We appreciate your careful evaluation of our manuscript and the valuable comments you have provided. Your detailed feedback has been instrumental in improving the clarity and robustness of our study. We have carefully addressed each of your comments in our revisions, and we appreciate your insights in helping us strengthen the manuscript!

**Comment#1:** Why is sika considered dominant over serow needs a bit more convincing explanation, the reference provided is based on occupancy, behavioral observation reference would be more convincing to subsequently then support by occupancy B coefficients. The size of Sika in comparison to Serow would be good to mention as well. Early on in the introduction it is mentioned that Serow are territorial while deer are not – is this territoriality behavior limited to conspecifics or extended towards other ungulates as well?

***Response:*** Thank you. This comment is related to reviewer 1’s **comment#11**. Please check

**Comment#2:** The RAI values need to be better used as when reduced to presence non-detection data for occupancy analyses – intensity (relative abundance) information is lost. An RAI bar chart with SE for different vegetation types could be used for depicting habitat use by deer and serow and if availability of vegetation types is available then preference could be computed and compared between deer and serow using indices like Ivlev’s index, etc. adding value to the ecological aspects of the MS.

***Response:*** Thank you for your constructive suggestion! However, to maintain statistical consistency, we believe that introducing a new analysis using RAI to assess habitat use is not appropriate. In this study, we used occupancy models to analyze habitat use and spatial segregation, ensuring a consistent methodological framework. It remains unclear whether the results from a single-species occupancy model would align with those based on RAI, and incorporating RAI as an additional analysis may compromise the consistency of interpretation. However, following your comments (**comment #7,8**), we have now incorporated model averaging to more rigorously assess the effectiveness of covariates, including vegetation type. We believe this revision sufficiently addresses your concern.

**Comment#3** (Line 154-): please explain what is considered as an independent event

***Response:*** In our study, we defined independent events in camera trap surveys based on the 30-minute rule proposed by O’Brien et al. (2003). According to your comments, we have added an explanation to clarify that consecutive detections of the same species at the same camera station were considered independent if they were at least 30 minutes apart as follows:

Added: “In our study, we defined independent events in camera trap surveys based on the 30-minute rule proposed by O’Brien et al. [35].” (Line 208-209)

**Comment#3** (Line 186-): The covariates for vegetation do not reflect in the results, either remove them or include them as they will provide lot of information regarding habitat use by the two species

***Response:*** Thank you for your valuable comment. We understand the importance of vegetation as a covariate in analyzing habitat use. However, in our results (single-species model), the vegetation model was not selected, which suggests that vegetation did not have a significant influence on the habitat use of Japanese serow and sika deer. Based on this outcome, we believe that removing or further emphasizing vegetation-related covariates may not be necessary. Nonetheless, we appreciate your insightful suggestion.

**Comment#4:** (Line 231-): Detected should be replaced by occupy as Psi corrects for detection bias?

***Response:*** You are correct! According to your comment, we have replaced "detected" with "occupy" as follows:

From: “The estimate of SIF(φ) < 1 is interpreted as indicating that the two species tend to be detected at the same site less frequently than expected under the assumption that the species select space independently (i.e., spatial segregation). The estimate of SIF(φ)>1 is interpreted as indicating that the two species tend to be detected at the same site more frequently than expected under the assumption that the species select space independently (i.e., spatial overlap).”

To: “The estimate of SIF(φ) < 1 is interpreted as indicating that the two species tend to be occupied at the same site less frequently than expected under the assumption that the species select space independently (i.e., spatial segregation). The estimate of SIF(φ)>1 is interpreted as indicating that the two species tend to be occupied at the same site more frequently than expected under the assumption that the species select space independently (i.e., spatial overlap).” (Line 297-302)

**Comment#5:** (Line 236-): Independent manner?

***Response:*** You are correct. According to your comment, we have replaced "in a dependent manner" with "in an independent manner" as follows:

From: “A SIF (φ) value of 1 indicates that the two species use a site in a dependent manner.”

To: “A SIF (φ) value of 1 indicates that the two species use a site in an independent manner.”

(Line 302-303)

**Comment#6:** In the table title replace Japanese deer with sika for consistency

***Response:*** According to your comment, we have replaced "Japanese deer" with "sika deer" in all Tables for consistency.

**Comment#7** (Line 261-266-): For both species and both seasons several competing models have substantial AIC support and therefore model averaged Beta coefficients should be the best way to interpret the results of single season occupancy.

**Comment#8**: The Beta coefficients of the best or model averaged for covariates needs to be reported and interpreted in the discussion section. These are important aspects of serow and deer ecology on how vegetation, slope and elevation effect their habitat use.

***Response:*** Thank you for your insightful comments. According to your comments, we have incorporated model-averaged Beta coefficients for single-season occupancy models. These coefficients provide a more comprehensive interpretation of habitat use by sika deer and Japanese serow in different seasons. Methods and Result section have changed as follows:

Method (added):

“The best model or model averaging was used to estimate coefficients and their 90% confidence intervals [43].” (Line 266-267)

Results:

From: “The best-fit single species occupancy model for serow included only the “year” for both summer and autumn (Table 2). However, in autumn, the null model ranked second and the third-ranked model included both "year" and "altitude" as competing models. For deer, the best-fit single species occupancy mode contained “year” in both summer and autumn, although the altitude was also selected as a competitive model in summer (Table 2). Considering these results, for the two-species models in summer, we included "year" as a covariate for both species.”

To “The best-fit single species occupancy model for serow included only the “year” for both summer and autumn (Table 1). However, in autumn, the null model ranked second and the third-ranked model included both "year" and "altitude" as competing models. The coefficients from the best model or the model-averaged estimates showed that “year” strongly influenced occupancy probability for Japanese serow, with higher occupancy in 2017. In autumn, a competing model included altitude, but its effect was small and uncertain, as its confidence interval widely spanned zero (Table 2). For deer, the best-fit single species occupancy mode contained “year” in both summer and autumn, although the altitude was also selected as a competitive model in summer (Table 1). The coefficients from the best model or the model-averaged estimates showed that “year” had a strong effect, with higher occupancy in 2017. In summer, altitude was included as a competing model, but its effect remained small and uncertain (Table 2). Considering these results, for the two-species models in summer, we included "year" as a covariate for both species.” (Line 336-348)

Furthermore, we have added “Based on the single occupancy model,” in the discussion (Line: 382) to clarify that vegetation, slope, and altitude did not have a significant effect on occupancy, as indicated by the model-averaged estimates.

**Comment#9** (Line 346-): "by partitioning their diets" citation required as no information on diet is present in this MS

***Response:*** We have revised the sentence to provide a more neutral phrasing and have included references to previous studies that describe the general dietary differences between these two species.

From: “Since there was no observable vegetation degradation and because food resources are abundant in Shirakawa Village, it appears that serow and deer are able to coexist, despite sharing the same habits, by partitioning their diets.”

To: “Since there was no observable vegetation degradation and because food resources are abundant in Shirakawa Village, it appears that serow and deer may be able to coexist, possibly due to differences in their dietary preferences [10,19].” (Line 465-468)

**Comment#10** (Line 369-): Local density would increase in pockets due to their rutting behavior, but overall densities should not change unless the study area attracts deer from neighboring areas during rut. Yes aggressive behavior would increase in these pockets.

***Response:*** Thank you. According to your comment, we have emphasized the increase in aggressive behavior within these local pockets.

From: “This increase in RAI values for deer during autumn is likely to 368 be related to their rutting

behavior when males form harems, leading to a temporary 369 increase in their density and aggressive behavior.”

To: “This increase in RAI values for deer during autumn is likely to be related to their rutting behavior, where males form harems, leading to localized density increases and intensified aggressive interactions.”

(line 484-486)

**Comment#11** (Line 383-): "without pronounced competition" can be omitted as the paper does not provide data to prove if the two species compete or not, only that they coexist without negative interactions.

***Response:*** According to your comment, we have deleted “without pronounced competition” as follows:

From: “In conclusion, our findings highlight that Japanese serow and Japanese deer in Shirakawa Village did not exhibit clear temporal or spatial segregation and appear to have the potential to coexist without pronounced competition”

To: “In conclusion, our findings highlight that Japanese serow and sika deer in Shirakawa Village did not exhibit clear temporal or spatial segregation and appear to have the potential to coexist.”

(Line 489-491)

**Comment#12** (Line 385-): "vital for them" can temper this down as your evidence says the contrary (mechanism for coexistence at high deer density.

***Response:*** According to your comment, we have toned down the statement as follows:

From: “This suggests that temporal segregation may be vital for them in order to coexist in areas where the deer population is increasing.”

To: “This suggests that temporal segregation may contribute to coexistence in areas where the deer population is increasing” (Line 503-504)
