# Supplementary material for: Temporal and spatial interactions in sympatric ungulates: Insights from Japanese serow and sika deer
Source: PLoS One. 2025 Aug 18;20(8):e0330171. doi: 10.1371/journal.pone.0330171 (PMC12360589; doi:10.1371/journal.pone.0330171)
Supplement: Attachment — Submitted filename: Response_to_Reviewers_auresp_2.docx [file pone.0330171.s003.docx]

**Additional Editor Comments**:
Thank you for submitting your manuscript to PLOS ONE. After careful consideration, we feel that it has merit but does not fully meet PLOS ONE’s publication criteria as it currently stands. Therefore, we invite you to submit a revised version of the manuscript that addresses the points raised during the review process.

After R1 the paper is much improved. Reviwer 2 indicate several points to modify such us resolving contradictions in abstract, more details in methodology and some context to tables. After this revision, the paper can be published.

***Response***: Thank you very much for the opportunity to revise our manuscript. We appreciate the positive feedback from the editor and reviewer. In response to Reviewer 2’s suggestions, we have carefully resolved the contradictions in the abstract, provided additional methodological details, and added necessary context and explanations to the tables. We believe these improvements have addressed the reviewer's concerns and enhanced the clarity and readability of our manuscript.

Thank you again for your valuable feedback and consideration.

**Reviewer #2:**

We sincerely appreciate the reviewer's time and effort in evaluating our manuscript, as well as their positive feedback!

<Abstract>

**Comment#1:** There is contradiction between the results and the conclusion in the abstract.

line 35 Although our study was limited by a small sample size, it revealed no clear temporal or spatial segregation between the species, suggesting that there is potential for coexistence in shared habitats without pronounced competitive conflict

and

line 43 Although our results are limited, they exhibit a similar trend to previous studies, suggesting that temporal segregation may play a crucial role in facilitating the sympatric coexistence of these ungulates.

If one reads the entire manuscript will understand but only from the abstract is confusing.

***Response:*** Thank you for the comment. We acknowledge the confusion caused by the contradictory statements in the abstract. According to your suggestion, we have revised line 43-44 of the abstract as follows:

From: “Although our results are limited, they exhibit a similar trend to previous studies, suggesting that temporal segregation may play a crucial role in facilitating the sympatric coexistence of these ungulates.”

To: “This seasonal adjustment indicates a context-dependent behavioural response that may serve to reduce temporal overlap and mitigate competition”

<Introduction>

**Comment#2:** Although at line 71 it is explained that the endemic Japanese serow (Capricornis crispus) (hereafter serow) and the more wide-ranging sika deer (Cervus nippon) (hereafter deer), there are several places where sika deer instead of deer appears. Please check carefully all the text.

***Response:*** We have carefully reviewed the entire manuscript and unified the terminology by consistently using "serow" and "deer" to refer to sika deer, as defined in line 71. However, we have retained the full species name "sika deer" in tables and figure captions for clarity and readability.

**Comment#3 (Line 234):** I doubt that the detection is constant. One possibility will be to keep occupancy constat and model detection and then use the best model for detection in modeling the occupancy. In the end there aren’t so many covariates.
**Comment#6 (Results):** I am surprised to see that a 90 % CI was used. I think you need to justify the choice given that 95% CI is more often used.

***Response:*** Thank you for your valuable comments. According to your comments, we have conducted model selection for both occupancy and detection probabilities under the constraint of including at most one categorical and one continuous variable for each component, taking into account the limited sample size (n = 39). In other words, each model included no more than a total of four covariates across occupancy and detection. Additionally, due to technical limitations, we reanalyzed the single-species models using the R package unmarked instead of PRESENCE.

Furthermore, we revised our inference approach by replacing the previously used 90% confidence intervals with standard p-values based on model-averaged estimates.

As a result of incorporating covariates into the detection component, some changes were observed in detection-related results. However, these changes did not affect the main conclusions of the study.

Please refer to the revised Methods and Results sections for details.

**Comment#4 (Line 241):** add a reference for the approach used

***Response:*** According to your comment, we have add the following reference.

Fuller, A. K., D. W. Linden, and J. A. Royle. 2016. Management decision making for fisher populations informed by occupancy modeling. Journal of Wildlife Management 80:794–802.

**Comment#5 (Line 297):** Not clear, please reformulate. I find the phrasing "tend to be occupied at the same site" a bit awkward.

***Response:*** According to your comment, we have revised the sentence at line 297 to improve clarity as follows:

From: “The estimate of SIF(φ) < 1 is interpreted as indicating that the two species tend to be occupied at the same site less frequently than expected under the assumption that the species select space independently (i.e., spatial segregation). The estimate of SIF(φ)>1 is interpreted as indicating that the two species tend to be occupied at the same site more frequently than expected under the assumption that the species select space independently (i.e., spatial overlap). A SIF (φ) value of 1 indicates that the two species use a site in an independent manner.”

To: “The estimate of SIF (φ) < 1 suggests that the two species co-occur less frequently than expected under the assumption of independence (i.e., spatial segregation). Conversely, SIF (φ) > 1 indicates that the two species co-occur more frequently than expected under independence (i.e., spatial overlap). A value of SIF (φ) = 1 implies that the two species occupy sites independently.”

**Comment#7 (Table 3,4):** All the abbreviation should be explained in the table caption. You did this for occupancy and detection but it is needed also for la A, B, a;

***Response:*** We appreciate the reviewer's suggestion. However, the subscripts such as "a" and "b" in parameters (e.g., pA, pB, ψBA, ψBa) represent conditional states of occupancy and detection probabilities and have already been clearly defined in the Methods section following the standard parameterization by MacKenzie et al. (2004, 2006). Repeating these definitions in the table caption would introduce redundancy and could lead to confusion by unnecessarily increasing complexity. To maintain clarity and readability, we explicitly define only the species abbreviations (A = sika deer; B = Japanese serow) in the caption, as these directly pertain to species identities and are essential for immediate interpretation.

**Comment#8 (Line 418-420):** the statement need some references

***Response:*** We have revised the wording to improve clarity. While we did not add new references, the sentence was rephrased to present the statement more cautiously and avoid the need for citation.

From: “The discrepancy between our findings and those of previous 439 studies might arise from factors,

such as varying degrees of human disturbance, different vegetation types, or resource availability.”

To: “Because the presence of predators, interspecific competition, and anthropogenic disturbance vary considerably among these study areas, such local factors may influence the activity patterns of Capricornis species.”
